# High-Density Surface Electromyography Excitation in Front vs. Back Overhead Press Prime Movers

**DOI:** 10.5114/jhk/205466

**Published:** 2025-12-10

**Authors:** Riccardo Padovan, Nicholas Toninelli, Stefano Longo, Gianpaolo Tornatore, Fabio Esposito, Emiliano Cè, Giuseppe Coratella

**Affiliations:** 1 Department of Biomedical Sciences for Health, Università Degli Studi di Milano, Milan, Italy.

**Keywords:** muscle activity, resistance exercise, strength training, eccentric, concentric

## Abstract

The current study compared the spatial excitation of the prime movers when performing the overhead press with the barbell passing in front (front-OHP) or behind the neck (back-OHP) using high-density surface electromyography (HD-sEMG). Fourteen resistance trained men performed both exercises within a non-fatiguing set with 8-RM as the external load. The HD-sEMG amplitude and the muscle excitation centroid of anterior deltoid, lateral deltoid, posterior deltoid, upper trapezius, pectoralis major and triceps brachii muscles were recorded during the ascending and the descending phase. During the ascending phase, the front-OHP showed a superior HD-sEMG amplitude of the anterior deltoid (ES = 1.03) and the pectoralis major (ES = 0.70). During the descending phase, the front-OHP showed a superior HD-sEMG amplitude of the anterior deltoid (ES = 1.00) and the pectoral major (ES = 0.74), while the back-OHP showed superior excitation of the posterior deltoid (ES = 1.11), the upper trapezius (ES = 0.72) and triceps brachii (ES = 2.01). During the ascending phase, the front-OHP showed a more medial centroid of the lateral deltoid (ES = 2.30). During the descending phase, the front-OHP showed a more lateral centroid of the lateral deltoid (ES = 0.87), whereas the centroid of the posterior deltoid (ES = 0.63) and the triceps brachii (ES = 0.68) was more medial. Additionally, the centroid was more cranial in the front- vs. the back-OHP for the posterior deltoid (ES = 1.10), while for the pectoralis major the centroid was more caudal (ES = 0.62). The front- and back-OHP appeared to provide different overall excitation in the prime movers. Moreover, distinct spatial excitation patterns were observed, making both exercises suitable for the training routine.

## Introduction

Resistance training uses targeted exercises to stimulate specific muscle groups, increasing muscle strength and size through adaptive responses to neural, mechanical, and metabolic stress (Duchateau et al., 2021; Morton et al., 2019; Suchomel et al., 2018). The biomechanical characteristics of each exercise represent the unique stimuli provided to the target muscles, influencing their excitation quantitatively and throughout their anatomical development (Vigotsky et al., 2018). Considering a given exercise, different variations can give different stimuli to the prime movers, allowing diversification of the overall training routine (Coratella, 2022). As for the muscles around the shoulder, it is possible to use the overhead press (OHP) exercise to stimulate the upper trapezius, triceps brachii, and pectoralis major, anterior, posterior deltoid, and lateral deltoid muscles (Coratella et al., 2022c). The OHP could be performed with different body positions (such as a seated position versus standing) (Saeterbakken and Fimland, 2013), using different external loads, e.g., a barbell, dumbbells, or kettlebells (Błażkiewicz and Hadamus, 2022; Padovan et al., 2024a), and when it comes to the barbell, moving it in front (front-OHP) or behind (back-OHP) the head (Coratella et al., 2022c). All these variations may provide the prime movers with different stimuli.

The excitation of the prime movers quantification using surface electromyography (EMG) may provide insights into the neural patterns of each muscle group (Vieira and Botter, 2021). Research has largely examined how prime movers are excited during different variations in many resistance exercises, such as squats (Clark et al., 2012; Coratella et al., 2021), the bench press (Cabral et al., 2022; Coratella et al., 2020; Gołaś et al., 2024), deadlifts (Andersen et al., 2019; Martín-Fuentes et al., 2020), rows (Fujita et al., 2020; Padovan et al., 2025), lat pull-downs (Andersen et al., 2014; Padovan et al., 2024b), lateral raises (Reinold et al., 2007), and biceps curls (Coratella et al., 2023; Marcolin et al., 2018). Among these, the bench press stands out as one of the most extensively investigated upper-body exercises in terms of EMG analysis. Multiple studies have explored how targeted training, load intensity, and muscle dominance affect the excitation of key prime movers such as the pectoralis major, the anterior deltoid, and the triceps brachii (Choi et al., 2023; Stronska et al., 2022; Stronska-Garbien et al., 2024). Furthermore, investigations into torque modulation and joint stabilization during pressing movements provide additional insights into the neuromuscular control involved (Krzysztofik et al., 2021). In contrast, the overhead press has received significantly less attention in EMG-based literature, despite being a fundamental overhead movement in both sports and resistance training. This gap underlines the importance of exploring shoulder and upper chest muscle recruitment in overhead pressing patterns. Notably, a previous work comparing front- and back-OHP variations reported greater excitation of the pectoralis major and the anterior deltoid in the front-OHP, and increased excitation of the posterior and the lateral deltoid in the back-OHP (Coratella et al., 2022c).

However, although the previous approach provides a quantitative analysis of the prime movers’ excitation, it does not specify how each prime mover is spatially stimulated. The development of high-density surface electromyography (HD-sEMG) has allowed for more detailed insights into the muscles’ role during a given motion (Vieira and Botter, 2021). HD-sEMG allows for spatial mapping of the EMG signal through a grid of electrodes rather than relying on a single electrode pair (Vieira and Botter, 2021), hence describing spatial information on muscle excitation patterns and offering a more comprehensive understanding of how different muscles contribute to many complex movements (Vieira and Botter, 2021). Indeed, HD-sEMG locates the excitation of the fascicles covered by the grid and provides mean spatial excitation defined as the muscle excitation centroid within the medio-lateral and the cranio-caudal plane (Vieira and Botter, 2021). So far, HD-sEMG has been used to investigate the difference in hamstring muscle excitation when performing various exercises (Hegyi et al., 2019) and to compare different bench press (Cabral et al., 2022), lat pull-down (Padovan et al., 2024b), and seated row variations (Padovan et al., 2025).

When examining the between-variation differences, distinguishing between the ascending and the descending phases offers the possibility to examine the neuromuscular stimuli derived mostly by the concentric and eccentric actions of the prime movers, respectively (Duchateau and Enoka, 2008, 2016; Enoka, 1996). Taking into account the acute (Duchateau and Enoka, 2016), short-term (Coratella and Bertinato, 2015), and sustained differences between concentric and eccentric stimuli (Coratella et al., 2022a, 2022b), separating the two phases may provide a deeper understanding of their distinct neural impact. To date, the only previous study that has examined the differences in muscle excitation when performing the front- or the back-OHP provided only a quantitative analysis of the muscle excitation (Coratella et al., 2022c). Therefore, the aim of the present study was to explore the spatial excitation patterns of the primary muscles during the front- and the back-overhead press (OHP). The results may be useful to further characterize each variation to be used as a specific stimulus in resistance training.

## Methods

### Study Design

This study was structured as a cross-over, within-subject design with repeated measures, following previously established protocols (Cabral et al., 2022; Coratella et al., 2022c; Padovan et al., 2024b). Participants attended three separate sessions. The first session allowed participants to familiarize themselves with the front- and back-OHP techniques and to establish electrode placements for all target muscles. In the second session, the 8-repetition maximum (8-RM) for each OHP variation was determined, with the order randomized (Padovan et al., 2024b). During the third session, maximum voluntary isometric contraction was first recorded for each muscle, followed by a minimum of 10 min of passive recovery. Afterward, surface electromyography (EMG) data collection took place during a non-exhaustive set of both OHP exercises, using a load corresponding to each participant's 8-RM and performing four repetitions to avoid fatigue. Each session was spaced at least three days apart, and participants were instructed to avoid any additional resistance training throughout the study period.

### Participants

A convenience sample of fourteen resistance-trained male participants (age 24.64 ± 3.82 years; body height 1.73 ± 0.06 m; body mass 75.33 ± 6.02 kg) was part of the study (Cabral et al., 2022; Hegyi et al., 2019; Mancebo et al., 2019; Padovan et al., 2024b). All participants had a minimum of three-year experience in resistance training. Eligibility criteria required participants to have no musculoskeletal injuries in the glenohumeral joint, the spine, or upper limbs in the past six months. Additionally, participants were asked to avoid caffeine, alcohol, and other stimulants for 24 hours prior to testing. The study received approval from the ethics committee of the University of Milan, Milan, Italy (approval code: CE 35/22; approval date: 05 May 2022) and was carried out in accordance with the Declaration of Helsinki (1964, with subsequent updates) for research involving human subjects. Participants were fully briefed on the study aims and procedures, provided written informed consent, and were informed that they could withdraw from the study at any stage.

### Exercises Technique

**Figure 1:**
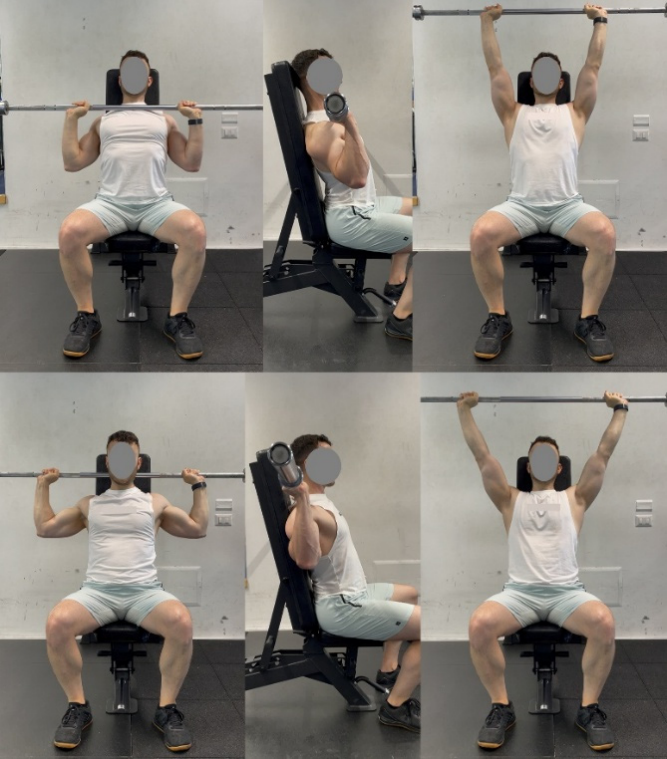
The technique for each exercise, described with a frontal and a lateral view of the start and a frontal view of the end of each movement: (above) front overhead press; (below) back overhead press.

Participants, sitting on a bench [Technogym, Gambettola (FC), Italy], performed both the front- and the back-OHP with an 80° inclined backrest, stabilizing the torso. An Olympic barbell (20 kilograms Vulcan Standard, Vulcan Strength Training System, Charlotte, NC, US) was used for both variations. For the front-OHP, participants held the barbell slightly wider than shoulder-width, with hands positioned just outside the deltoids. They lifted the barbell from chest level to full elbow extension, positioning it above and slightly behind the head. Forearms were to remain perpendicular to the floor throughout the lift, and an observer monitored the movement visually. This positioning created a curved, C-shaped bar path in the front-OHP. For the back-OHP, participants used hand spacing that allowed a 90° angle at the shoulder and elbow joints (Figure 1). The barbell was raised starting below the occipital bone to a complete extension above the head, with a relatively straight bar path (Paoli et al., 2010). To accommodate the barbell’s rearward path, the trunk was inclined slightly backward (90°) while still supported in the lumbar region. At the end of the ascending phase, participants held the bar in an isometric position for 0.5 s before beginning the descent, completing the full range of motion (ROM) as described in standard resistance exercise protocols (Coratella, 2022). For both exercises, each phase—ascending and descending—was performed over 2 s, marked by a metronome, with an isometric pause of around 0.5 s. Participants visually received feedback on timing throughout each lift (Padovan et al., 2024b).

### 8-RM Protocol

The 8-RM was evaluated using the previously described technique, following established protocols (Coratella et al., 2022c; Padovan et al., 2024a). Participants first completed a standardized warm-up, performing three sets of 15 repetitions of the OHP using progressively heavier self-selected weights which were individually selected. Identifying the 8-RM load involved gradually raising the weight amount until participants were not able to conclude the 8^th^ repetition during the ascending phase, signifying failure (Kompf and Arandjelović, 2016). A minimum of three minutes of passive resting isolated every participant’s attempt, who also received verbal encouragement to maximize effort in each trial, per standardized guidelines. This procedure was carried out for both OHP exercises in randomized order.

### Muscle Excitation Detection

The signal coming through EMG was collected utilizing semi-reusable high-density electrode grids following a configuration of a 13 x 5 arrangement (GR08MM1305 model, 8 mm inter-electrode spacing, OT Bioelettronica, Turin, Italy) during both OHP exercises. Muscles on the dominant side were monitored, including the anterior, lateral, and posterior deltoid, the upper trapezius, the triceps brachii, and the pectoralis major. For the anterior, lateral, and posterior deltoid and the triceps brachii, grids were aligned to the muscle fibres and placed lengthwise (Barbero et al., 2012). The grids were laid down perpendicularly to muscle fibres on the pectoralis major and the upper trapezius (Vieira and Botter, 2021).

The area of innervation was not taken into account for pectoralis major and upper trapezius muscles according to the “Atlas of Muscle Innervation Zones” (Barbero et al., 2012), whilst the positioning of the grid was also placed in the innervation area for anterior, lateral, and posterior deltoid and triceps brachii muscles, in line with previous research (Campanini et al., 2022; Merletti and Muceli, 2019; Padovan et al., 2024b; Rodriguez-Falces et al., 2013). For these muscles, the positioning of the grid was carefully located to minimize unwanted signals from adjoining muscles (Vieira and Botter, 2021). Grid placement specifics included positioning for the anterior deltoid approximately 2 cm below the clavicle along a line between the coracoid process and deltoid tuberosity (Barbero et al., 2012).

Considering the lateral deltoid, the grid was aligned in the middle of the lateral epicondyle and the acromion, above the deltoid tuberosity (Barbero et al., 2012). The grid of the posterior deltoid was positioned on the higher portion, about 2 cm from the acromion side edge (Barbero et al., 2012). The upper trapezius grid was positioned on the higher section, about 2 cm sideways to the prominent vertebra (Barbero et al., 2012), while the grid of the pectoralis major was set about 2 cm below the clavicle (Barbero et al., 2012). Considering the triceps brachii, the application of the grid was done over the long head, at around one-third of the distance from the acromion to the medial epicondyle of the humerus (Barbero et al., 2012). Conductive cream (ac cream, Spes Medica s.r.l., Genoa, Italy) was applied to the cavities of the electrode grid to maintain consistent contact with the skin. Skin preparation included shaving and abrasion with an abrasive paste (Nuprep, Weaver and Company, Colorado, USA). EMG data were collected in monopolar configuration at a sampling rate of 2048 Hz, with a gain of 200 (Casolo et al., 2023), using an electromyography system (EMG-USB2+, OT Bioelettronica, Turin, Italy) (Merletti et al., 2001). Reference electrodes were placed on the wrist (ground electrode) and on the acromion (high-density grid references).

Once the grids were applied, each participant performed maximum voluntary isometric contractions for each targeted muscle following guidelines from the “Atlas of Muscle Innervation Zones” (Barbero et al., 2012). For the anterior deltoid, standing participants had one arm by their side and the elbow flexed, applying isometric force by lifting the arm upward against resistance (Barbero et al., 2012). For the lateral deltoid, participants stood with the arm abducted to 45° and the elbow flexed, performing an isometric contraction by abducting the shoulder against resistance at the elbow (Barbero et al., 2012). For the posterior deltoid, participants generated maximal force by driving the arm, positioned at 90° abduction, backward against resistance at the elbow (Barbero et al., 2012). Concerning the upper trapezius, participants were seated with the arm abducted at 90° and were instructed to elevate the scapula and the clavicle’s acromial end against a downward, immovable resistance (Barbero et al., 2012). The activation for the pectoralis major involved participants attempting horizontal adduction with the arm and the shoulder flexed at 90° against a fixed resistance (Barbero et al., 2012). Lastly, for the triceps brachii, participants stood with one arm close to their body, applying isometric force to extend the elbow at a 45° angle (Barbero et al., 2012). Each muscle contraction was performed three times, with each attempt lasting 5 s, and a recovery period of 3 min was provided between attempts (Coratella et al., 2023). Operators offered standardized verbal encouragement to promote maximal effort during each attempt. Following a 10-min passive recovery, participants engaged in a non-exhaustive set of the front and the back OHP, with the order randomized. A 3-min rest interval separated each set, and the load was set to the previously determined 8-RM. Each set included four repetitions to minimize fatigue and maintain technique consistency. The tempo of 2 s for both the ascending and the descending phase was regulated by a metronome, with an operator monitoring to ensure no changes in movement speed.

### Muscle Excitation Centroid

Using electrode grids in EMG measurements facilitates the analysis of muscle excitation’s spatial distribution across the grid area. To quantify this distribution, the root mean square (RMS) values were used to calculate the barycenter along the vertical (y-axis) and horizontal (x-axis) axes, expressed in mm relative to the grid’s coordinates. This calculation, known as the central locus of activation, describes how muscle excitation is spatially distributed (Watanabe et al., 2012). The centroid, represented by the barycenter of EMG amplitude values along the grid’s rows and columns, was identified for the anterior deltoid, lateral deltoid, posterior deltoid, upper trapezius, pectoralis major, and triceps brachii muscles (Gallina et al., 2013).

### Data Analysis

EMG data were recorded in a monopolar mode with a gain of 200 (Casolo et al., 2023) and, using a 12-bit analog-to-digital converter with a 5-volt dynamic range, the data were digitized at 2048 Hz. A bandpass filter was applied, spanning 20–400 Hz (Merletti et al., 2001). The root mean square (RMS) was used to characterize the EMG signals in the time domain. For maximum voluntary isometric contractions, a 1-s interval was analyzed. For each exercise, RMS values were calculated and averaged over the central second of both the upward and downward phases. Synchronization between EMG signals and each exercise phase was facilitated by a digital camera (iPhone 12, 12MP resolution, 1080p, 60fps, Apple, California) mounted on a tripod. This setup allowed precise marking of phase transitions in the EMG analysis (Cabral et al., 2022). To ensure consistent execution, the first repetition of each set was excluded from the EMG analysis (Marri and Swaminathan, 2016). The EMG RMS values for each muscle in each exercise were subsequently normalized (nRMS) to the muscle's peak voluntary isometric excitation (Coratella et al., 2023; Padovan et al., 2024b).

**Figure 2:**
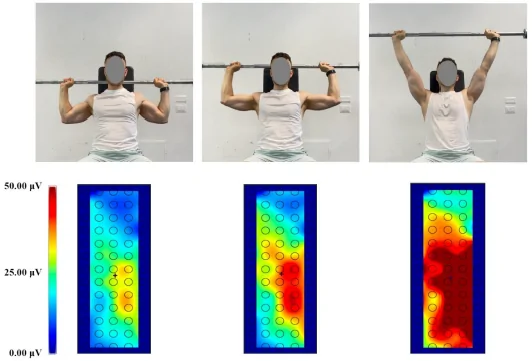
A typical spatial map of the muscle excitation for the anterior deltoid during the back overhead press. The upper panel shows three distinct positions, and the corresponding spatial excitation is reported below for each position. The centroid is represented by “+”.

Using MATLAB version R2023B (The MathWorks, Inc, Natick MA, USA), a color map of muscle excitation was created from the RMS values of all 64 grid channels (Figure 2). To obtain color maps, the monopolar EMG signals were bandpass filtered (20–400 Hz) and differentiated into 12 or 4 single-differential EMG signals, depending on the orientation of the fibers relative to the matrix orientation (Cabral et al., 2022). Subsequently, their RMS amplitude was computed. Only active channels, identified as those detecting surface EMG signals with an RMS amplitude exceeding 70% of the maximum amplitude across the electrode matrix, were included in the subsequent analysis (Cabral et al., 2022). The 70% amplitude threshold was selected due to its proven effectiveness in accurately identifying channels located above highly active fibers (Vieira et al., 2010). The number and the interquartile range of active channels were then computed to evaluate the spread of the RMS amplitude distribution. Additionally, the barycentre of these active channels, defined as the weighted average of their coordinates, was calculated to assess where along the matrix cranio-caudal and medial-lateral axis the EMG amplitude was most strongly represented. The location of the centroid was determined by translating the position of each electrode in the matrix into x- and y-coordinates, measured in mm along the two axes (Padovan et al., 2024b). The central second of the ascending and descending phases was analyzed to exclude the transition moments between the phases from the analysis.

### Statistical Analyses

Statistical analyses were conducted using SPSS software version 28.0 (IBM, Armonk, NY, USA). Data normality was checked with the Shapiro-Wilk test, which confirmed that all distributions were normal. Descriptive statistics for the 14 participants were presented as mean (SD). To examine differences in the normalized RMS (nRMS) and centroid positions between the front- and the back-OHP across the descending and ascending phases, a two-way repeated-measures ANOVA was performed for each muscle. Bonferroni correction was applied for multiple comparisons, and results were reported as mean differences with 95% confidence intervals (95% CI). Statistical significance was set at α < 0.05. The magnitude of main effects and interactions was determined using partial eta squared (ηp^2^) and classified as trivial (≤0.009), small (0.010–0.059), medium (0.060–0.139), or large (≥0.140) (Cohen, 1988). Pairwise comparisons were presented as means with 95% confidence intervals and effect sizes (ES) based on Cohen’s *d*. Effect sizes were interpreted following Hopkins et al.’s (2009) guidelines: trivial (0.00–0.19), small (0.20–0.59), moderate (0.60–1.19), large (1.20–1.99), and very large (≥2.00).

## Results

The average 8-RM load was 36.8 (5.1) kg for the front-OHP and 33.2 (3.3) kg for the back-OHP (*p* < 0.01; ES = 1.11, 0.42 to 1.77).

**Figure 3:**
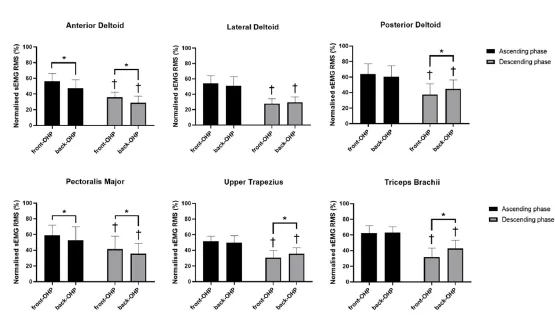
The mean (SD) of the normalized root mean square (nRMS) recorded during the ascending and the descending phase of the front (front-OHP) and the back overhead press (back-OHP) is shown for each muscle. Besides front vs. back overhead press differences, nRMS was greater during the ascending than the descending phase in all exercises. * p < 0.05 vs. back-OHP. ⴕ p < 0.05 vs. ascending phase

Figure 3 displays the nRMS recorded from all muscles during the ascending and descending phases of the front- and the back-OHP. An interaction between the exercise and the phase was observed for the nRMS in the lateral deltoid (F = 6.548, *p* = 0.024, ηp^2^ = 0.335), posterior deltoid (F = 34.014, *p* < 0.001, ηp^2^ = 0.723), upper trapezius (F = 14.154, *p* = 0.002, ηp^2^ = 0.521) and triceps brachii muscles (F = 27.522, *p* < 0.001, ηp^2^ = 0.679), while no interaction was observed in anterior deltoid (F = 1.029, *p* = 0.329, ηp^2^ = 0.073) and pectoralis major mucles (F = 0.035, *p* = 0.855, ηp^2^ = 0.003). In the ascending phase, the nRMS was higher in the anterior deltoid during the front-OHP compared to the back-OHP (8.79%, 3.85% to 13.73%; ES = 1.03, 0.36 to 1.67) and also in the pectoralis major (6.53%, 1.11% to 11.95%; ES = 0.70, 0.10 to 1.27), while lateral deltoid, posterior deltoid, upper trapezius and triceps brachii muscles had similar excitation (*p* > 0.05). During the descending phase, the nRMS was greater in the front- than the back-OHP in anterior deltoid (6.98%, 2.93% to 11.02%; ES = 1.00, 0.34 to 1.63) and pectoralis major muscles (5.87%, 1.31% to 10.42%; ES = 0.74, 0.14 to 1.33), while greater in the back-OHP in posterior deltoid (7.33%, 3.51% to 11.14%; ES = 1.11, 0.42 to 1.77), upper trapezius (5.09%, 0.99% to 9.19%; ES = 0.72, −0.11 to 1.23) and triceps brachii muscles (10.72%, 7.64% to 13.80%; ES = 2.01, 1.07 to 2.93). No difference was recorded in the lateral deltoid. The ascending phase showed higher nRMS values than the descending phase across all muscles during both the front- and the back-OHP (*p* < 0.05 for all comparisons).

**Figure 4:**
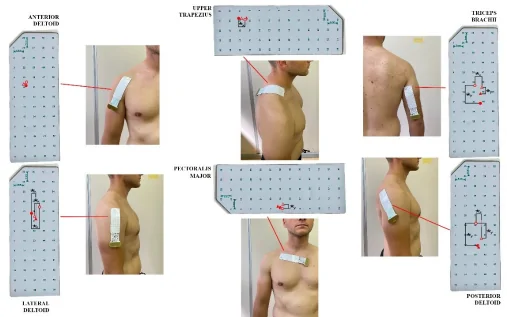
Spatial muscle excitation for the muscles analyzed is shown. The grids are visualized as positioned on each muscle. The upward and downward direction indicates a cranial and caudal shift on the vertical plane, respectively; the rightward and the leftward shift indicates a lateral and a medial shift on the horizontal plane, respectively. The front overhead press (front-OHP) is represented graphically by filled circles (⚫) for the ascending and empty circles (🔘) for the descending phase. The back overhead press (back-OHP) is represented graphically by filled triangles (▲) for the ascending and empty triangles (△) for the descending phase. ✱y: p < 0.05 comparing the centroid on the vertical y-axis; ✱x: p < 0.05 comparing the centroid on the horizontal x-axis

Figure 4 illustrates the average horizontal and vertical coordinates of the centroid for each muscle during the ascending and descending phases of both the front- and the back-OHP. An interaction between the exercise and the phase was noted for the horizontal axis in the lateral deltoid (F = 61.051, *p* < 0.001, ηp^2^ = 0.824), the upper trapezius (F = 7.092, *p* = 0.020, ηp^2^ = 0.353) and the triceps brachii (F = 6.409, *p* = 0.025, ηp^2^ = 0.330), while no interaction was identified in the anterior deltoid (F = 0.938, *p* = 0.351, ηp^2^ = 0.067), the posterior deltoid (F = 4.210, *p* = 0.061, ηp^2^ = 0.245) and the pectoralis major (F = 1.162, *p* = 0.301, ηp^2^ = 0.082). During the ascending phase, the centroid was more medial in the front- vs. the back-OHP in the lateral deltoid (3.20%, 0.23% to 6.17%; ES = 2.30, 1.27 to 3.31). No medial-lateral differences in the centroid were found for anterior deltoid, posterior deltoid, pectoralis major, upper trapezius, or triceps brachii muscles (*p* > 0.05). However, during the descending phase, the centroid was positioned more laterally in the lateral deltoid during the front-OHP compared to the back-OHP (3.97%, 1.33% to 6.61%; ES = 0.87, 0.24 to 1.47), while the posterior deltoid (4.32%, 0.35% to 8.28%; ES = 0.63, 0.43 to 1.19) and the triceps brachii (5.74%, 0.88% to 10.59%; ES = 0.68, 0.09 to 1.26) exhibited the opposite behavior, with the centroid positioned more medially. No medial-lateral differences in the centroid were observed for anterior deltoid, pectoralis major, or upper trapezius muscles (*p* > 0.05). The centroid was positioned more laterally in the lateral deltoid (6.37%, 4.77% to 7.97%, ES = 2.30, 1.27 to 3.31) and the upper trapezius (5.13%, 0.26% to 9.99%, ES = 0.61, 0.03 to 1.17) during the descending phase compared to the ascending phase of the front-OHP. No additional between-phase differences were observed (*p* > 0.05).

An interaction between the exercise and the phase was observed for the vertical axis in posterior deltoid (F = 5.830, *p* = 0.031, ηp^2^ = 0.310), pectoralis major (F = 5.867, *p* = 0.031, ηp^2^ = 0.311) and triceps brachii muscles (F = 17.117, *p* = 0.001, ηp^2^ = 0.568), while no interaction was observed in anterior deltoid (F = 1.189, *p* = 0.295, ηp^2^ = 0.084), lateral deltoid (F = 0.883, *p* = 0.365, ηp^2^ = 0.064) and upper trapezius muscles (F = 0.463, *p* = 0.508, ηp^2^ = 0.034). During the ascending phase, no cranio-caudal differences were observed in the centroid between the front- and the back-OHP (*p* > 0.05). In the descending phase, the centroid was positioned more cranially in the posterior deltoid during the front-OHP compared to the back-OHP (12.49%, 2.86% to 22.12%; ES = 1.10, 0.42 to 1.76), whereas the pectoralis major exhibited the opposite trend, with the centroid located more caudally (3.08%, 0.22% to 5.94%; ES = 0.62, 0.04 to 1.12). Additionally, the centroid was more cranial in the posterior deltoid (18.27%, 8.71% to 27.83%; ES = 1.10, 0.42 to 1.76) and the triceps brachii (14.83%, 8.29% to 21.36%; ES = 1.31, 0.57 to 2.02) during the descending phase compared to the ascending phase of the front-OHP. The triceps brachii (5.49%, 0.51% to 10.46%, ES = 0.64, 0.05 to 1.20) showed similar behavior in the back-OHP. No further between-phase difference was found (*p* > 0.05).

## Discussion

This study examined the excitation of the prime movers involved in the front- and the back-OHP, utilizing high-density surface EMG for the first time and distinguishing between the ascending and descending phases. The key findings were that the front-OHP demonstrated greater external loads compared to the back-OHP. During the ascending phase, the anterior deltoid and the pectoralis major exhibited higher excitation in the front-OHP, while in the descending phase, the posterior deltoid, the upper trapezius, and the triceps brachii showed greater excitation in the back-OHP, with anterior deltoid and pectoralis major muscles still displaying greater excitation in the front-OHP. On the horizontal plane, during the ascending phase, the front-OHP induced a medial shift in the centroid for the lateral deltoid, while during the descending phase, it induced a medial shift for the posterior deltoid and the triceps brachii. On the vertical plane, during the descending phase, the front-OHP induced a cranial shift in the centroid for the posterior deltoid and a caudal shift for the pectoralis major. These different excitation patterns appear to be associated with the front- and the back-OHP. Before discussing the different muscles’ excitation, some preliminary considerations may be useful to contextualize the results. In the first instance, the absolute external load was generally greater during the front- than the back-OHP, and it is known that the load itself can influence muscle excitation, with greater loads eliciting greater RMS amplitudes (Looney et al., 2016). Second, the biomechanical characteristics related to the different exercise techniques can either enhance or limit the ability to lift heavier loads (Coratella et al., 2021), and this should be taken into account when interpreting the role of each primary muscle. In this regard, both the different trajectories (C-shaped for the front-OHP and straight for the back-OHP) (Coratella et al., 2022c) and the different between-hand distance may have led to the muscles operating at different lengths with repercussions on the sEMG signal amplitude (Vigotsky et al., 2018). Moreover, although both variations share the same ending point, we speculate that the external load travels a greater distance during the front-OHP compared to the back-OHP, deriving from the lower starting point of the former, as dictated by the technique. Combined with the possibly longer trajectory, this may result in higher movement velocity for the same cadence to perform both the ascending and the descending phase. Since nRMS amplitude tends to be higher during quicker movements (Frost et al., 2008), this should also be considered when comparing the two. Lastly, the present procedures were conducted using HD-sEMG, examining a larger muscle area in comparison to a pair of single electrodes (Vieira and Botter, 2021) so that the mean muscle excitation can be mediated by a larger muscle portion. Thus, the possible differences with the only previous study on the topic may derive from the different methods (high-density vs. single electrodes), loads (8-RM vs. 80%-1RM), or the participants’ training experience (three-year trained vs. competitive bodybuilders) between the studies (Coratella et al., 2022c).

During the ascending phase, the anterior deltoid and the pectoralis major showed greater excitation in the front-OHP, while no further difference was found. Considering the different barbell trajectories, the possible greater humerus flexion in the front- than the back-OHP (Coratella et al., 2022c) may have elicited the role of the anterior deltoid and the pectoralis major. As for upper trapezius, lateral and posterior deltoid, and triceps brachii muscles, the RMS amplitude was similar comparing the front- and the back-OHP. While this might be expected for the lateral deltoid as responsible for a similar shoulder abduction (Escamilla et al., 2009), for the upper trapezius as responsible for similar scapular elevation and rotation (Escamilla et al., 2009), and the triceps brachii since the movement probably requires similar elbow extension to press the weight overhead (Coratella et al., 2022c), this might appear unexpected for the posterior deltoid, given the greater external humerus rotation needed to let the barbell pass behind the neck in the back- than the front-OHP (Coratella et al., 2022c). However, such a dissimilarity was observed throughout the descending phase, where maybe such an external rotation was more emphasized to stabilize the barbell backward (Coratella et al., 2022c). Moreover, during the descending phase, the anterior deltoid and the pectoralis major were still more excited in the front- than the back-OHP, and no difference was observed for the lateral deltoid, as during the ascending phase. In contrast, upper trapezius and triceps brachii muscles showed higher excitation during the back-OHP. As for the upper trapezius, this may depend on the need for greater stabilization when the weight is behind the head, preventing scapular winging and maintaining the posture (Coratella et al., 2022c; Padovan et al., 2024b). Regarding the triceps brachii, this may derive from greater action in the shoulder joint stabilization when the arm is abducted posteriorly (Landin et al., 2018).

The novel approach using HD-sEMG provides a more qualitative assessment of muscle excitation (Vieira and Botter, 2021), highlighting the placement of the mean excitation within each muscle. It should be noted that the interpretation of the centroid placement determined by HD-sEMG for each muscle may not follow the muscle anatomical planes, especially when referring to the medio-lateral plane, since it is based on each matrix reference axis. Furthermore, the placement of the centroid along the horizontal medio-lateral and the vertical cranio-caudal plane is related to different mechanisms (Vieira and Botter, 2021). Indeed, while the former provides an overview of the spatial excitation within the muscle and between the main fascicles (Vieira and Botter, 2021), the latter mostly indicates the shift of the innervation zone during dynamic contractions (Mancebo et al., 2019; Vieira et al., 2017), together with the conduction velocity (Vieira and Botter, 2021) not examined here.

Considering the matrix positioning in medio-lateral plane transverse to the fascicles, information obtained is related to the different involvement between different parallel fascicles (Vieira and Botter, 2021), thus fascicles with the greater excitation tended to shift the centroid in their direction. Comparing the front- and the back-OHP, the centroid of the lateral deltoid was more medial, i.e., more posterior, during the ascending phase of the front- vs. the back-OHP, as also observed for the posterior deltoid, i.e., more medial, during the descending phase. Both may derive from the greater elongation of the posterior fascicles of the lateral and the posterior deltoid (Fridén and Lieber, 2001; Lorne et al., 2001), which might have increased the mean EMG amplitude when rotating internally the humerus internal during the front-OHP (Padovan et al., 2024b). For possibly similar mechanisms deriving from the elongation of the fascicles, the lateral deltoid exhibited a more lateral centroid during the descending phase in the front- vs. the back-OHP. Still focusing on the medial-lateral axis, the triceps brachii had its laterally shifted centroid in the back- vs. the front-OHP during the descending phase, probably due to the fact that the prominent humerus internal rotation could have favored the elongation and consequent excitation of medial fascicles (Padovan et al., 2024b), which is in line with the above. Throughout the descending phase, the lateral deltoid exhibited a more lateral placement of the centroid in the front- vs. the back-OHP, in contrast to the ascending phase. The explanation could reside in the different fascicle elongation during each phase. Indeed, during the descending phase, lateral deltoid fascicles experience non-uniform elongation due to the anterior load positioning (Coratella et al., 2022c; Padovan et al., 2024b), with the posterior segment slightly shortened as the shoulder moves into a more extended position and the anterior undergoing greater elongation. Since the HD-sEMG centroid reflects the average position of fascicle excitation, the possible greater excitation of the anterior portion may have led to a centroid lateral shift.

As to the cranio-caudal plane, the matrix positioning was parallel to the fascicles, allowing the detection of EMG signals in the same fascicles along their length (Vieira and Botter, 2021). As such, the centroid is affected by the innervation zone sliding due to the fascicle shortening or elongation (Mancebo et al., 2019; Vieira et al., 2017). Consequently, the signal propagates longitudinally from the innervation zone, increasing its amplitude (Mancebo et al., 2019). Considering the pectoralis major, the centroid shifted more caudally in the front- compared to the back-OHP during the descending phase. In the front-OHP, the possible greater ROM due to the lower barbell could have elongated the fascicles towards their origin at the clavicle level with the consequent innervation zone shift, resulting in more mean caudal excitation (Jiroumaru et al., 2014). As for the posterior deltoid, the centroid shifted more caudally in the back- vs. the front-OHP during the descending phase. With regard to the matrix vertical axis in line with the fascicle’s orientation, it seems that the front-OHP shifted the excitation versus the scapula, probably due to the fascicle shortening and the consequent innervation shift towards the muscle origin. Comparing the ascending and the descending phase within every exercise, the upper trapezius had its centroid shifted laterally in the descending vs. the ascending phase during the front-OHP. Considering the anatomical action of the upper trapezius during the front-OHP, this results in a scapula upward rotation during the ascending phase, in opposition to the downward rotation during the descending phase (Johnson et al., 1994). As a consequence, the fascicles with the origin at the cranial vertebrae (more lateral) were more elongated during the downward rotation of the scapula compared to the fascicles with a caudal vertebrae origin (more medial) (Johnson et al., 1994). Given the matrix transversal placement to the fascicles, this may have reflected their elongation, with a consequent shift of the centroid in a more lateral position throughout the descending phase. Moreover, the front-OHP exhibited a more caudal centroid for the lateral deltoid, while the triceps brachii showed a centroid shifted cranially in the descending vs. the ascending phase in both the front- and the back-OHP. Since the matrix was placed in line with the fascicles, it is possible that the elbow flexion may have elongated the fascicles with the consequent caudal shift of the innervation zone, resulting in a more cranially centroid placement (Mancebo et al., 2019).

The present study has some limitations that should be acknowledged. First, the results were influenced by the combination of the technique used, the selected load, and the participants' sports backgrounds. Altering any of these factors could impact the outcomes. Regarding technique, the front-OHP typically involves a greater ROM than the back-OHP due to differing movement trajectories and mobility demands. Comparing these exercises with a similar ROM (e.g., limiting the depth in the front-OHP) might yield different results. Second, examining the excitation of additional muscles, such as the sternocostal head of the pectoralis major and the same muscles on the contralateral side, could provide further insights and facilitate a more comprehensive discussion. Finally, using different load ranges (e.g., 6- and 10-RM) during data collection might offer additional insights into muscle excitation.

## Conclusions

In conclusion, the current study found different muscle excitations between the front- and the back-OHP. Quantitively, the front-OHP induced greater excitation of the anterior deltoid and the pectoralis major, while in the back-OHP, greater excitation of triceps brachii, upper trapezius, and posterior deltoid muscles was mostly visible during the descending phase. As for a more qualitative analysis of the muscle excitation through the centroid, medio-lateral differences were found in the lateral and posterior deltoid, as well as triceps brachii muscles, while cranio-caudal differences were observed in the posterior deltoid and the pectoralis major. Overall, the front- and the back-OHP do not induce equivalent muscle excitation and should be used to target the muscles surrounding the shoulders in different ways.

The front- and the back-OHP seem to produce varying overall excitation in the primary muscles. Here are some practical considerations. Besides the diversity in the prime movers’ stimuli, both exercises are not equivalent in terms of the possibility of being performed effectively and safely. Indeed, the back-OHP demands higher gleno-humeral joint mobility to be performed, as discussed in previous literature (Coratella et al., 2022c; Padovan et al., 2024b). Individuals with limited shoulder mobility might need to create additional movements to let the barbell pass behind the neck. Moreover, a previous study has observed a possibly greater shoulder instability due to the “high-five” position in the back-OHP, highlighting the need for proper technique and adequate mobility for safe execution (Kolber et al., 2013). The authors also noted that the results could be influenced by false positive evaluations, suggesting that the relationship may not be straightforward (Kolber et al., 2013). Instead of completely disregarding the backward variation, it may be more beneficial to gradually train individuals to familiarize themselves with the shoulder capacity required and then perform the back-OHP initially with very light loads (Coratella et al., 2022c). As such, the increased stimuli provided to the posterior shoulder muscles may reinforce them to contrast some kyphotic postures, especially in sedentary people (Coratella et al., 2022c).
